# Urban-Rural Differences in Sarcopenia Prevalence and Nutritional Risk Factors: The NHANES (2001–2002 and 2011–2014)

**DOI:** 10.1093/geroni/igaa057.870

**Published:** 2020-12-16

**Authors:** Jason Aziz, Kieran Reid, John Batsis, Roger Fielding

**Affiliations:** 1 Tufts University, Boston, Massachusetts, United States; 2 Dartmouth College, Lebanon, New Hampshire, United States

## Abstract

Background: Older adults living in rural areas experience health inequities compared to their urban counterparts. These include comorbidities, poor diet and physical inactivity; known risk factors for sarcopenia. No studies examining urban-rural differences in the prevalence of sarcopenia and slow gait speed among older adults in the United States exist. Objective: To compare the prevalence of sarcopenia and slow gait speed between urban and rural older adults living in the United States. As a secondary aim, we examined relationships between rural residency, total energy and total protein on gait speed and grip strength. Methods: We performed a secondary data analysis of two cohorts in the continuous NHANES (2001-2002 and 2011-2014), using gait speed or grip strength data, along with urban-rural status, dietary, examination, questionnaire and demographic data in older (≥ 60 yrs.) adults. Results: The prevalence of GripBMI weakness was higher in urban vs. rural participants (27.4% vs. 19.2%), whereas their absolute grip strength was lower (31.75(±0.45) vs. 33.73(±0.48)). Total energy, total protein and relative protein intakes were similar between urban and rural participants. Total energy intake was associated with gait speed and grip strength. Conclusions: Older adults living in urban areas of the United States, were weaker compared to their rural counterparts. Rural residency was not associated with gait speed or grip strength. Total energy intake was associated with slower gait speed but higher grip strength. This report is the first to examine urban-rural differences in sarcopenia and slow gait speed in older adults living in the United States.

